# Supplementary material for: CD46 targeted 212Pb alpha particle radioimmunotherapy for prostate cancer treatment
Source: J Exp Clin Cancer Res. 2023 Mar 11;42:61. doi: 10.1186/s13046-023-02636-x (PMC10007843; doi:10.1186/s13046-023-02636-x)
Supplement: Supplementary file 1 — Additional file 1: Table S1. Hematology parameters of acute (day-14) and long-term (day-90) toxicity studies. RBC: Red blood cell; WBC: White blood cell; PLT: Platelet; HCT: Hematocrit test (proportion of RBC in blood); HGB: Hemoglobin; Neu: Neutrophil; Lym: Lymphocyte; Mon: Monocyte; and Eos: Eosinophil. Mean ± SD, n = 5. Table S2. Blood clinical chemistry tests for liver and kidney function for acute and long-term toxicity studies. Figure S1. The calibration curve of Pb-Arsenazo (Pb-AAIII) spectrophotometric assay for determining the TCMC chelator number per antibody. The standard curve of UV (upper panel) is constructed by plotting the absorbance at 656 nm against Pb2+ concentration at various dilutions from a stock solution of Pb(II)-AA(III). The stock solution was prepared in a 0.15 M NH4OAc buffer at pH 7.0, containing 10 mM AAIII and 4.83 mM Pb(II).. The titration curve (bottom panel) is constructed by plotting the change of UV absorbance at 656 nm (%) against the mass of TCMC added to 100 µl of the Pb(II)-AA(III) complex solution. The solution had a Pb2+ concentration of 192 µM. Figure S2. Therapeutic effects of a single dose 212Pb-TCMC-YS5 in the PC3 subcutaneous CDX model. Tumor growth of individual mice within 55 days post treatment of 212Pb-TCMC-YS5 (normalized against tumor volume at day 0). Red: Study group receiving 212Pb-TCMC-YS5. Blue: Control group receiving unlabeled YS5 antibody only. Figure S3. BLI images of the PC3-Luc orthotropic CDX model treated by a single dose 212Pb-TCMC-YS5 (top), and the cold YS5 control (bottom). Two animals died in the control group on day-19 post treatment and were not imaged. Figure S4: Change (days post injection) of actual tumor volume of individual PDX mouse treated with a single dose of 0.37MBq (10 μCi) and 0.74 MBq (20 μCi) of 212Pb-TCMC-YS5. [file 13046_2023_2636_MOESM1_ESM.docx]

**Supplemental materials**

**CD46 targeted ^212^Pb alpha particle radioimmunotherapy for prostate cancer treatment**

Jun Li, Tao Huang, Jun Hua, Qiong Wang, Yang Su, Ping Chen, Scott Bidlingmaier , Allan Li, Zhongqiu Xie , Anil P. Bidkar, Sui Shen, Weibin Shi, Youngho Seo, Robert Flavell, Daniel Gioeli, Robert Dreicer, Hui Li, Bin Liu , Jiang He

**List of supplemental data:**

**Table S1:** Hematology parameters of acute (day-14) and long-term (day-90) toxicity studies.

**Table S2:** Blood clinical chemistry tests for liver and kidney function for acute and long-term toxicity studies.

**Figure S1:** The calibration curve of Pb-Arsenazo spectrophotometric assay for determining TCMC number per antibody.

**Figure S2:** Therapeutic effects of a single dose ^212^Pb-TCMC-YS5 in the PC3 subcutaneous CDX model.

**Figure S3:** BLI images of the PC3-Luc orthotropic CDX model treated by a single dose ^212^Pb-TCMC-YS5.

**Figure S4:** Change of actual tumor volume of individual PDX mouse treated with a single dose of 0.37MBq (10 μCi) and 0.74 MBq (20 μCi) of ^212^Pb-TCMC-YS5.

**Table S1**

Hematology parameters of acute (day-14) and long-term (day-90) toxicity studies. RBC: Red blood cell; WBC: White blood cell; PLT: Platelet; HCT: Hematocrit test (proportion of RBC in blood); HGB: Hemoglobin; Neu: Neutrophil; Lym: Lymphocyte; Mon: Monocyte; and Eos: Eosinophil. Mean ± SD, n = 5

|  | 14 days | | 90 days | |
| --- | --- | --- | --- | --- |
|  | ^212^Pb-TCMC-YS5 | No treatment | ^212^Pb-TCMC-YS5 | No treatment |
| RBC (10^6^/µL) | 7.62±0.66 | 7.99±0.23 | 6.68±0.57 | 8.15±0.08 |
| WBC (10^3^/µL) | 4.15±0.94 | 5.28±1.72 | 6.23±3.07 | 5.39±1.29 |
| PLT (10^3^/µL) | 1366.50±208.76 | 1515.20±107.71 | 1494.83±219.88 | 1625.00±160.97 |
| HCT | 35.98±3.51 | 39.52±1.14 | 31.47±4.13 | 39.02±1.46 |
| HGB (g/dL) | 12.58±1.24 | 13.48±0.49 | 11.05±1.11 | 13.18±0.56 |
| Neu (%) | 26.35±9.38 | 13.40±1.94 | 17.98±3.80 | 19.44±5.74 |
| Lym (%) | 68.35±11.45 | 82.62±3.36 | 76.13±6.11 | 74.80±8.10 |
| Mon (%) | 4.02±1.87 | 1.62±0.90 | 2.92±1.60 | 2.44±1.79 |
| Eos (%) | 1.23±0.42 | 2.34±1.05 | 2.88±1.86 | 3.26±1.46 |

**Table S2**

Blood clinical chemistry tests for liver and kidney function for acute and long-term toxicity studies. Mean ± SD, n = 5.

|  | 14 days | | 90 days | |
| --- | --- | --- | --- | --- |
|  | ^212^Pb-TCMC-YS5 | No treatment | ^212^Pb-TCMC-YS5 | No treatment |
| Albumin (µg/mL) | 34.8±3.8 | 26.4±10.9 | 30.1±5.5 | 30.4±5.3 |
| Creatinine (µmol/L) | 18.7±5.6 | 15.1±5.8 | 17.3±1.2 | 14.7±1.4 |
| Blood Urea Nitrogen (mmol/L) | 15.8±3.2 | 9.4±4.1 | 13.3±4.6 | 13.2±4.6 |
| Alanine transaminase (ng/mL) | 15.9±2.0 | 13.6±0.9 | 16.3±1.1 | 15.5±1.2 |
| Aspartate transaminase (ng/mL) | 113.5±11.3 | 91.4±32.6 | 119.3±29.3 | 106.3±29.7 |

**
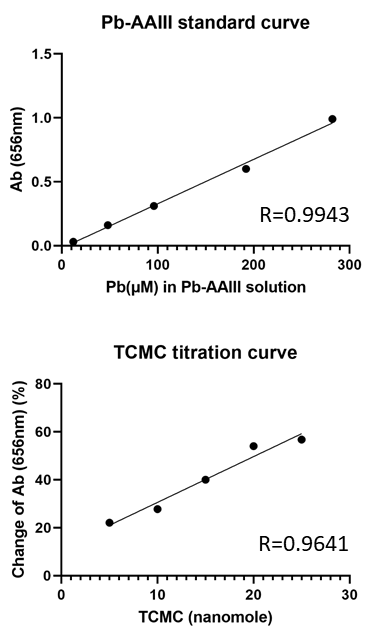
**

**Figure S1**: The calibration curve of Pb-Arsenazo (Pb-AAIII) spectrophotometric assay for determining the TCMC chelator number per antibody. The standard curve of UV (upper panel) is constructed by plotting the absorbance at 656 nm against Pb2+ concentration at various dilutions from a stock solution of Pb(II)-AA(III). The stock solution was prepared in a 0.15 M NH4OAc buffer at pH 7.0, containing 10 mM AAIII and 4.83 mM Pb(II).. The titration curve (bottom panel) is constructed by plotting the change of UV absorbance at 656 nm (%) against the mass of TCMC added to 100 µl of the Pb(II)-AA(III) complex solution. The solution had a Pb2+ concentration of 192 µM.

**
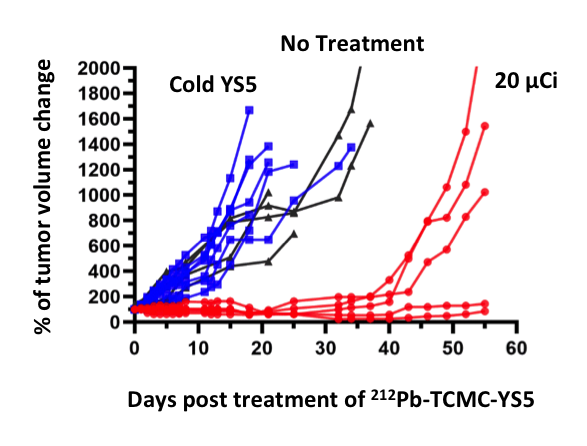
**

**Figure S2:** Therapeutic effects of a single dose ^212^Pb-TCMC-YS5 in the PC3 subcutaneous CDX model. Tumor growth of individual mice within 55 days post treatment of ^212^Pb-TCMC-YS5 (normalized against tumor volume at day 0). Red: Study group receiving ^212^Pb-TCMC-YS5. Blue: Control group receiving unlabeled YS5 antibody only.


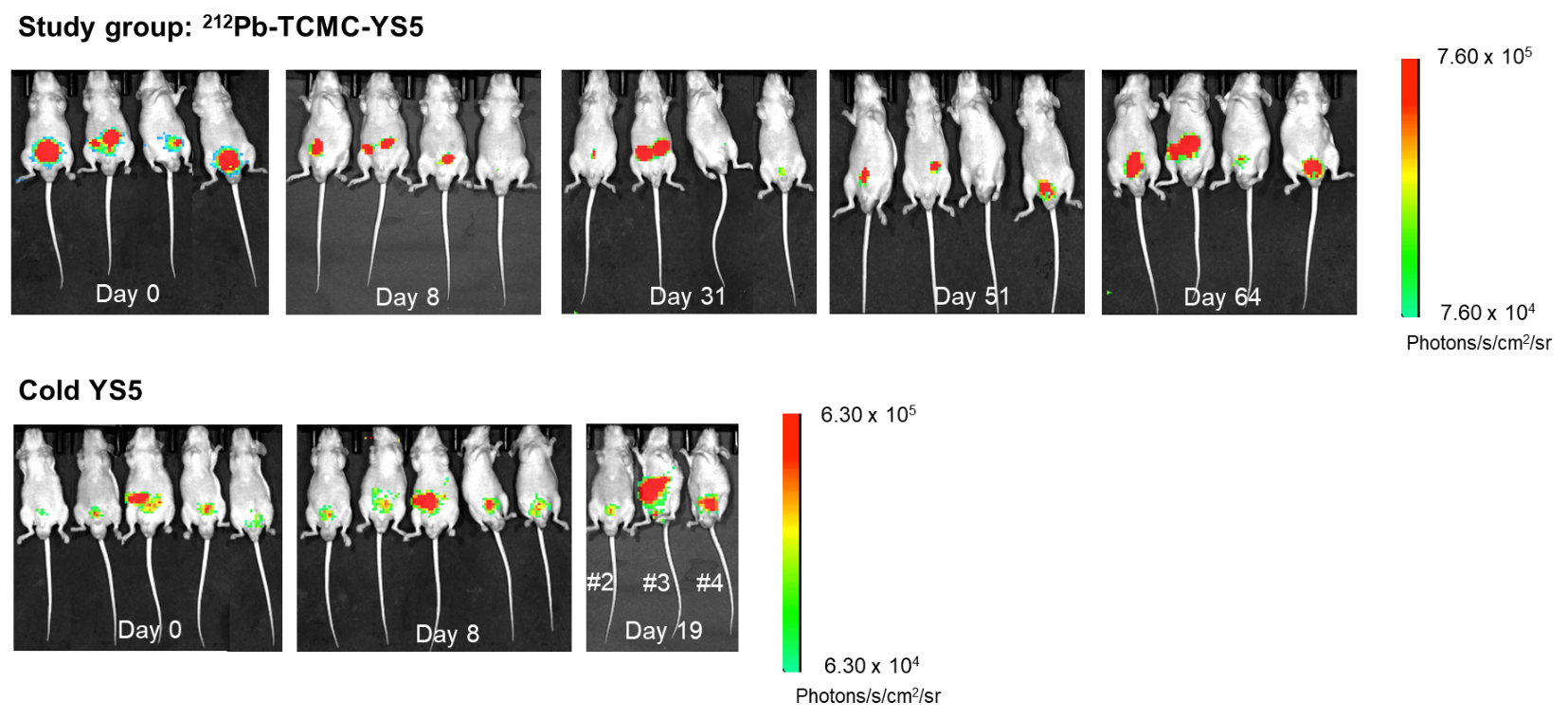


**Figure S3:** BLI images of the PC3-Luc orthotropic CDX model treated by a single dose ^212^Pb-TCMC-YS5 (top), and the cold YS5 control (bottom). Two animals died in the control group on day-19 post treatment and were not imaged.


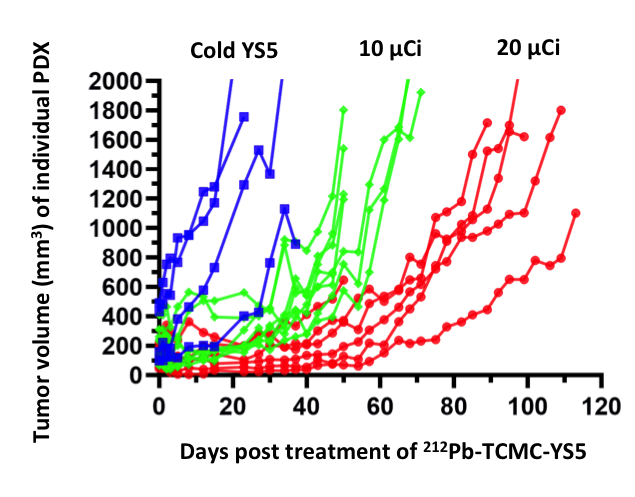


**Figure S4:** Change (days post injection) of actual tumor volume of individual PDX mouse treated with a single dose of 0.37MBq (10 μCi) and 0.74 MBq (20 μCi) of ^212^Pb-TCMC-YS5.
